# Human papillomavirus infection and use of oral contraceptives

**DOI:** 10.1038/sj.bjc.6600971

**Published:** 2003-05-27

**Authors:** J Green, A Berrington de Gonzalez, J S Smith, S Franceschi, P Appleby, M Plummer, V Beral

**Affiliations:** 1 Cancer Research UK Epidemiology Unit, University of Oxford, Gibson Building, Radcliffe Infirmary, Oxford OX2 6HE, UK; 2 Unit of Field and Intervention Studies, International Agency for Research on Cancer, 150 cours Albert Thomas, F-69372 Lyon cedex 08, France

**Keywords:** human papillomavirus, oral contraceptives, cervix cancer

## Abstract

Human papillomavirus (HPV) infection is thought to be a necessary but not sufficient cause of most cases of cervical cancer. Since oral contraceptive use for long durations is associated with an increased risk of cervical cancer, it is important to know whether HPV infection is more common in oral contraceptive users. We present a systematic review of 19 epidemiological studies of the risk of genital HPV infection and oral contraceptive use. There was no evidence for a strong positive or negative association between HPV positivity and ever use or long duration use of oral contraceptives. The limited data available, the presence of heterogeneity between studies and the possibility of bias and confounding mean, however, that these results must be interpreted cautiously. Further studies are needed to confirm these findings and to investigate possible relations between oral contraceptive use and the persistence and detectability of cervical HPV infection.

Infection with certain ‘high-risk’ types of the human papillomavirus (HPV) is thought to be a necessary but not sufficient cause of most cases of cervical cancer (IARC Working Group on the Evaluation of Carcinogenic Risks to Humans, 1999; Walboomers *et al*, 1999). In recent analyses of data from case–control and cohort studies, an increased risk of cervical cancer with increasing duration of oral contraceptive use was confirmed both in all women and in HPV positive women (Moreno *et al*, 2002; Smith *et al*, 2003). This suggests that long duration use of oral contraceptive may influence the development of cervical cancer in women infected with HPV, however in interpreting these results it is important to know whether such use of oral contraceptives is itself associated with HPV infection. We have reviewed available evidence from epidemiological studies on any relation between the use of oral contraceptives and genital HPV infection.

## METHODS

Studies were identified through a search of MEDLINE (1966–August 2002) using the search terms ‘papillomavirus’, search restricted to human studies (pre-1994) or ‘papillomavirus, human’ (1994–2002) and ‘risk factors’, and supplemented by references from identified studies. Two studies then in press (Anh *et al*, 2003; Shin *et al*, 2003) were obtained from groups reporting relevant data at the *International Papillomavirus Conferences*, 2000–2002. Studies were eligible for inclusion in this review if they had: (i) at least 200 subjects with normal cervical cytology or from a population with ‘mixed’ normal and abnormal cytology but at relatively low risk for cytological abnormality, such as general population surveys or routine screening or gynaecological clinics (studies of women attending colposcopy clinics or with conditions such as HIV infection, likely to be associated with a substantially increased risk of cervical abnormality, were excluded); (ii) any measure of genital HPV infection as the outcome; (iii) information on oral contraceptive use; and (iv) relative risk estimated for HPV positivity in oral contraceptive users *vs* never users either adjusted for age, or calculated for age-matched or age-restricted subjects. The most adjusted relative risk available was used for analysis. No restriction was placed on the language of publication. The term ‘HPV positivity’ is used to reflect the fact that what is measured by the various tests for HPV is detectable HPV rather than, necessarily, underlying HPV infection. ‘High-risk’ (oncogenic) and ‘low-risk’ (nononcogenic) HPV types were as defined in each eligible study; all studies included types 16 and 18 in high-risk and types 6 and 11 in low-risk groups, but studies varied with regard to the number and classification of other types.

In most studies, the term ‘oral contraceptive’ was used irrespective of the dose or formulation of contraceptive pills and it was not possible to distinguish in these studies between the use of combined or progestogen-only pills. It is likely, however, that the large majority of oral contraceptive users had used preparations containing combined oestrogen and progestogen (Collaborative Group on Hormonal Factors in Breast Cancer, 1996; IARC Working Group on the Evaluation of Carcinogenic Risks to Humans, 1999).

For analysing the effect of duration of oral contraceptive use, results were grouped as ‘short duration’ (less than 5 years use), ‘medium duration’ (5–9 years use), and ‘long duration’ (10 or more years use), with published results allocated to the most appropriate group. For studies that had more than one data point within a duration category, data were combined using the method of generalised least squares taking into account the correlations between the relative risks being combined (Berrington and Cox, in press). In this analysis, current use of oral contraceptives is use described as ‘current’ or use within the past 12 months and past use is use described as ‘past’ or use that ceased more than 12 months in the past.

The results are summarised graphically in the figures, which show relative risks for HPV positivity in users of oral contraceptives compared with never users. Studies are listed in order of date of publication, earliest first. Black squares indicate relative risks, the area of each square being proportional to the amount of statistical information contributed, with horizontal lines indicating 95% confidence intervals. The method of empirically weighted least squares (Cox and Snell, 1989) was used to test for heterogeneity between relative risks.

## RESULTS

Overall, 19 eligible studies were identified, with data on HPV status and oral contraceptive use for a total of 20 509 women (Kjaer *et al*, 1990, 1997; Ley *et al*, 1991; Karlsson *et al*, 1995; Burk *et al*, 1996; Kotloff *et al*, 1998; Giuliano *et al*, 1999, 2001, 2002; Deacon *et al*, 2000; Richardson *et al*, 2000; Rousseau *et al*, 2000; Sellors *et al*, 2000; Lazcano-Ponce *et al*, 2001; Peyton *et al*, 2001; Berrington *et al*, 2002; Molano *et al*, 2002; Moreno *et al*, 2002; Anh *et al*, 2003; Shin *et al*, 2003). Details of the studies are given in Table 1

**Table 1:** Epidemiological studies eligible for this review of the relation between human papillomavirus (HPV) positivity and use of oral contraceptives (oral contraceptives)

**Study**	**Country**	**Year**	**Design**	**Number of subjects with HPV and oral contraceptive data**	**Cytology**	**HPV measure**	**HPV type**	**% HPV positive in study**	**RR adjusted for^a^**	
									**Sexual partners**	**Other**
Kjaer *et al* (1990)	Denmark, Greenland	1986	C/s population	1114	Mixed	FISH	Any	14	Yes	Area, cytology, age at first oral contraceptive use
							High risk: 16,18	12	Yes	Area, cytology
							Low risk: 6,11	7	Yes	Area, parity
Ley *et al* (1991)	USA	1989	C/s college	467	Mixed	PCR MY09/11	Any	46	Yes	Race
Karlsson *et al* (1995)	Sweden	1989	C/s population	526	Mixed	PCR MY09/11, GP5/6	Any	22	No	—
Burk *et al* (1996)	USA	1992–94	C/s college	602	Mixed	PCR MY09/11,Southern blot	Any	28	No	—
Kjaer *et al* (1997)	Denmark	1991–93	C/s population	956	Normal	PCR GP5/6	Any	15115	Yes	Parity, condom use
							High risk: 16, 18+		Yes	Chlamydia
							Low risk: 6,11,uc		Yes	
Kotloff *et al* (1998)	USA	1992–93	C/s college	414	Mixed	PCR MY09/11	Any	35	Yes	Race, condom use, parity, smoking, STDs, age at first intercourse
Giuliano *et al* (1999)	USA	1992–95	C/s clinic	971	Mixed	HC	High risk: 16,18+	13	Yes	Place of birth, marital status
Deacon *et al* (2000)	UK	1987–93	C/s controls^b^	384	Mixed	PCR MY09/11	Any	47	No	—
Richardson (2000)	Canada	1992–93	C/s college	375	Mixed	PCR MY09/11	Any	23	No	—
							High risk: 16, 18+	12		
							Low risk: 6,11+,uc	13		
Rousseau *et al* (2000)	Brazil	1993+	Cumulative c/c	765	Mixed	PCR MY09/11	High risk: 16, 18+	N/a		No -
							Low risk: 6, 11+, uc	N/a		
Sellors *et al* (2000)	Canada	1998–99	C/s clinic	954	Mixed	HC ll	High risk: 16, 18+	13	Yes	Marital status, smoking
Giuliano *et al* (2001,2002)	USA/Mexico	1997–98	C/s clinic	2031	Mixed	PCR MY09/11	Any	14	No	—
							High risk: 16, 18+	12		
							Low risk: 6,11+	3		
Lazcano-Ponce *et al* (2001)	Mexico	1996–99	C/s population	1340	Normal	PCR L1	Any	13	Yes	Marital status, parity, age at first intercourse, socioeconomic status
							High risk: 16, 18+	10		
							Low risk: 6,11+	3		
Peyton *et al* (2001)	USA	1996–2000	C/s clinic	3671	Mixed	PCR MY09/11	Any	39	Yes	Race, education, income, marital status, parity, diaphragm use, STDs
							High risk: 16, 18+	27		
							Low risk: 6,11+	15		
Berrington *et al* (2002)	UK	1984–91	C/s controls^b^	393	Mixed	Serology for E7 proteins	High risk: 16,18	11	Yes	Smoking
Molano *et al* (2002)	Colombia	1993–95	C/s population/clinic	1845	Normal	PCR GP5+/6+	Any	15	Yes	Education, parity, condom use, smoking, age at first intercourse
							High risk: 16, 18+	11		
							Low risk: 6, 11+	3		
Molano *et al* (2002)	Multicentre	1985–97	C/s controls^b^	1916	Normal	PCR MY09/11,GP5+/6+	Any	13	No	Centre
							High risk: 16, 18+	8		
							Low risk: 6, 11+	5		
Anh *et al* (in press)	Vietnam (Ho Chi Minh)	1997	C/s clinic	922	Mixed	PCR GP5+/6+	Any	11	Yes	Education, smoking, parity, abortion, HSV
Shin *et al* (in press)	Korea	1999–2000	C/s population	863	Mixed	PCR GP5+/6+Serology for VLPs	Any	10	No	—
							High risk: 16, 18+	20		

OC=oral contraceptive; RR=relative risk; C/s=cross-sectional study; c/c=case–control study; population=general population; clinic=family planning or general gynaecological clinic; FISH=filter *in situ* hybridisation; HC=hybrid capture; VLP=virus-like particle; N/a=not applicable; STD=sexually transmitted disease; HSV=herpes simplex virus. HPV types: 16, 18=types 16 and 18 only; 16,18+=16,18 and other high-risk types; 6,11=types 6 and 11 only; 6,11+=6,11 and other low-risk types; uc=uncharacterised types.

a All RR adjusted for age, or based on age-restricted subjects. Not all analyses adjusted for all variables shown.

b These studies use control women from case–control studies of cervical cancer as their subjects.

. The study by Moreno *et al* (2002) provided a pooled analysis of data on control subjects from eight case–control studies of cervical cancer. In two other studies, the subjects were also controls from case–control studies of cervical cancer (Deacon *et al*, 2000; Berrington *et al*, 2002). All studies measured prevalent HPV positivity, that is, the numbers of women testing positive at one point in time, with no distinction between recent and persistent infection; in the study by Rousseau *et al* (2000), women becoming HPV positive within the 12 months of study follow-up were also classed as HPV positive. In four studies (Kjaer *et al*, 1997; Lazcano-Ponce *et al*, 2001; Molano *et al*, 2002; Moreno *et al*, 2002), all subjects had normal cervical cytology; in the other studies, the proportion of women with normal cytology ranged from 89 to 99%. In all but one study, HPV DNA was detected in cervical specimens by polymerase chain reaction (PCR)-based (15 studies) or hybridisation methods; in the study by Berrington *et al* (2002) and in part of the study by Shin *et al* (2003), high-risk (genital) HPV types were detected by serology.

The following analyses are based on the data presented in published reports for the duration of use of oral contraceptives and for ever, current and past use. In many studies, the information provided was insufficient to allow inclusion of the study in all analyses.

Figure 1

**Figure 1:**
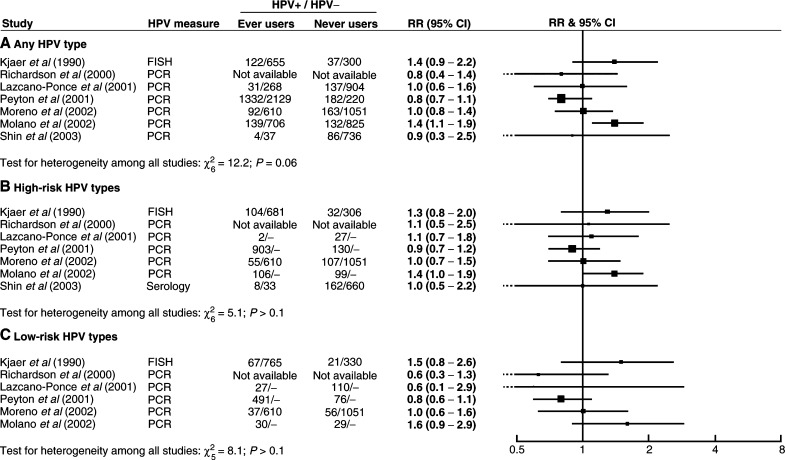
Relative risk (RR) and 95% confidence interval (CI) for (**A**) any type, (**B**) high-risk and (**C**) low-risk HPV positivity in ever users *vs* never users of oral contraceptives.

shows the relation between HPV positivity and ever use of oral contraceptives in the seven studies that presented these results. Results are shown for any HPV type, and separately for high-risk and low-risk HPV types. There was considerable variation between studies in the relative risks for HPV positivity in ever users *vs* never users of oral contraceptives, and no evidence for a strong positive or negative effect overall. Summary relative risks for ever *vs* never use have not been calculated as we do not feel that they would adequately reflect the variability in results between studies. The results of a statistical test for heterogeneity are included in this and subsequent figures for information; it should be borne in mind that such tests generally have a low sensitivity (Greenland, 1987) and should not be used as the sole criterion of important heterogeneity between studies.

**Figure 2:**
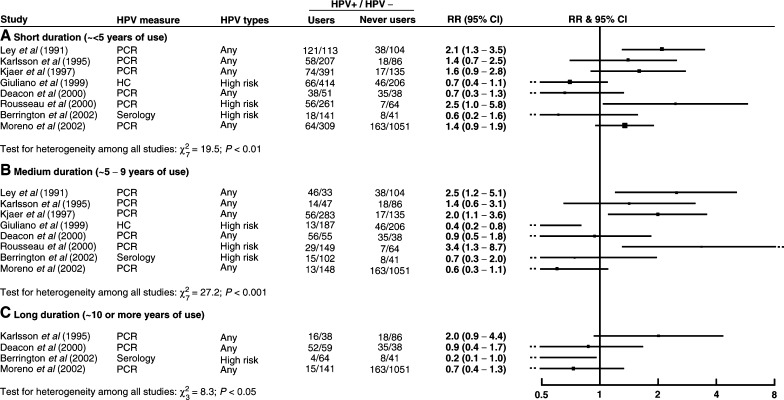
Relative risk (RR) and 95% confidence interval (CI) for HPV positivity for (**A**) short-, (**B**) medium- and (**C**) long-duration users *vs* never users of oral contraceptives.

Information on duration of use was available from eight studies, of which only four included long duration. There was no clear evidence for an effect of duration of oral contraceptive use on the risk of HPV positivity (Figure 2

). There was no consistency between studies in apparent trend of HPV positivity with duration of use, with individual studies showing increased, decreased or unchanged risk of HPV positivity with increasing duration of oral contraceptive use. The lack of information on long duration use and the variability between study results mean that, again, calculation of summary relative risks would not be appropriate.

**Figure 3:**
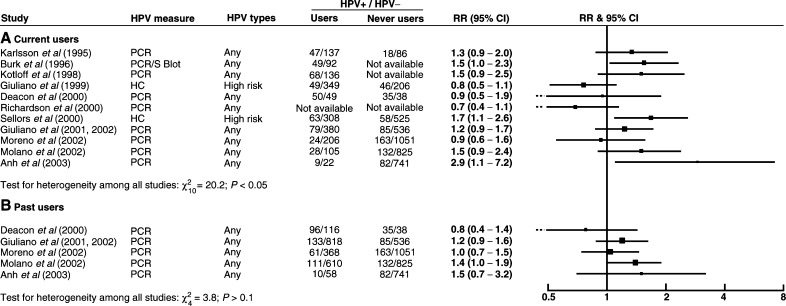
Relative risk (RR) and 95% confidence interval (CI) for HPV positivity in (**A**) current and (**B**) past users *vs* never users of oral contraceptives.

Relative risks for HPV positivity in relation to current and past use of oral contraceptives are shown in Figure 3

. Again there was considerable variability between study results and no clear evidence for a strong positive or negative association between oral contraceptive use and HPV positivity.

**Figure 4:**
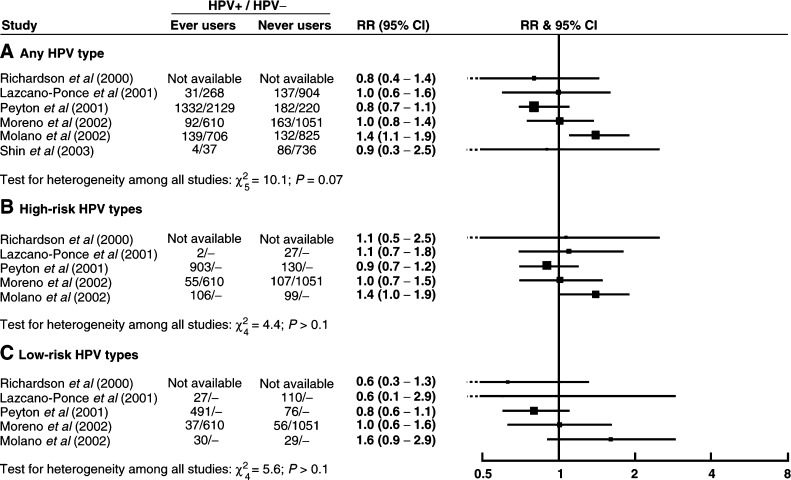
Relative risk (RR) and 95% confidence interval (CI) for (**A**) for any type, (**B**) high-risk and (**C**) low-risk HPV positivity in ever users *vs* never users of oral contraceptives, restricted to PCR studies.

Lack of information severely limited attempts to investigate possible sources of heterogeneity within this analysis. Figures 4

, 5

**Figure 5:**
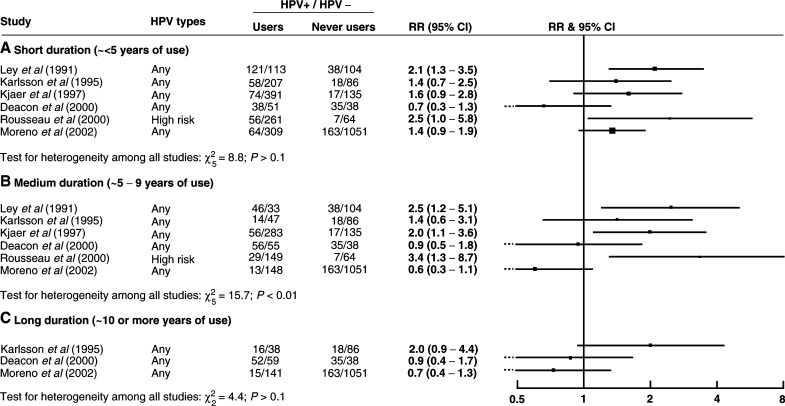
Relative risk (RR) and 95% confidence interval (CI) for HPV positivity for (**A**) short-, (**B**) medium- and (**C**) long-duration users *vs* never users of oral contraceptives, restricted to PCR studies.

and 6

**Figure 6:**
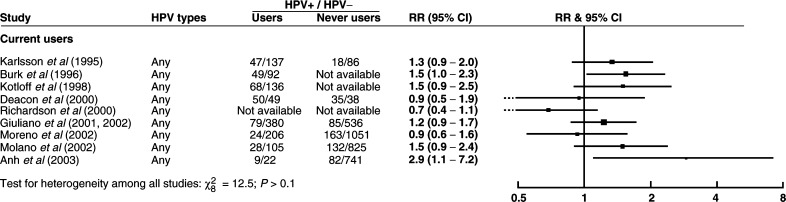
Relative risk (RR) and 95% confidence interval (CI) for HPV positivity in current users *vs* never users of oral contraceptives, restricted to PCR studies.

show the results for ever use, duration of use and current use in the 15 studies that used PCR methods to detect HPV (all of the studies with data on past use had used PCR). There were no obvious differences in the results of this analysis compared to the results of the analysis of all 19 studies, although restriction to PCR-based studies was associated with some reduction in statistical heterogeneity between studies. There were too few studies using non-PCR measures of HPV positivity to allow a direct comparison of PCR and non-PCR studies. The results from 10 of the 19 studies had been adjusted for the lifetime number of sexual partners. Restriction to these studies did not materially alter the findings of this review, and there remained considerable variability between studies. The number of studies available for each analysis was, however, very limited (four studies with information on ever use of oral contraceptives; four with information on short and medium duration of use but only one with information on long duration use; five with information on current and two with information on past use). Lack of information meant that it was not possible to attempt to assess the importance of the type of population (normal or mixed cytology) or of adjustment for factors such as socioeconomic status, smoking and reproductive factors.

## DISCUSSION

We found no evidence from available data for a strong positive or negative association between the use of oral contraceptives and the simultaneous or later detection of HPV on the cervix. In particular, there was no clear evidence for an effect of duration of oral contraceptive use on HPV positivity.

The present results must, however, be interpreted with caution. Limited information was available for many of the analyses, and there was considerable heterogeneity in study design and in results. There are also a number of potentially important sources of bias and confounding within the available data.

The prevalence of cervical HPV positivity is strongly related to age, with younger women having a higher prevalence, especially of high-risk HPV types (Melkert *et al*, 1993; Jacobs *et al*, 2000; Chan *et al*, 2002); and so is oral contraceptive use. Although this review has included only results adjusted for age (or, in two studies, restricted to women within a narrow age range), the degree of adjustment in most studies was probably insufficient to account fully for potential confounding. Most studies used 5–10 year age bands for adjustment and there is evidence that HPV prevalence may vary widely within such bands (Ley *et al*, 1991; Melkert *et al*, 1993). Residual confounding by age is particularly important in the analyses of the effects of duration of use of oral contraceptives, as short-duration users will tend to be younger than long-duration users.

Sexual activity is an important potential confounding factor in the studies considered here. The number of male sexual partners, in particular, is a major risk factor for cervical HPV infection (IARC Working Group on the Evaluation of Carcinogenic Risks to Humans, 1995) and oral contraceptive use is related to sexual behaviour (Swan and Brown, 1981; Svare *et al*, 1997), although it seems likely that the nature of this relation varies substantially with age, time and place (Oddens and Lehert, 1997; Spinelli *et al*, 2000). We found no obvious difference in results between studies with adjustment for the lifetime number of sexual partners and those without in this review, but the information available was insufficient to address this question fully.

The relation between duration of oral contraceptive use and HPV positivity may be critical in understanding the epidemiological and aetiological relations between oral contraceptives and HPV infection. Related to this is the question of the possible effects of oral contraceptive use on the persistence and detectability of HPV infection. The studies reviewed here provide very limited information on long duration of oral contraceptive use, and (with one exception) deal only with prevalent HPV positivity measured at varying times in relation to oral contraceptive use. Insufficient information was available to allow investigation of possible differences between the risks for high-risk and low-risk HPV positivity in the analyses of duration of use or of current or past use of oral contraceptives, although patterns of infection may differ between high-risk and low-risk HPV types (Elfgren *et al*, 2000; Jacobs *et al*, 2000).

For completeness, we have included in this review studies using different methods of detection of HPV. Differences in sensitivity and specificity between PCR-based and non-PCR-based tests could introduce bias as misclassification of subjects as HPV positive or negative will tend to attenuate a true association between a risk factor and HPV status. In this review, restriction to PCR-based studies did not materially alter the results. Differential misclassification, in which the sensitivity or specificity of an HPV test is itself related to the risk factor being studied, in this case to oral contraceptive use, remains a possible source of bias within studies using any method of HPV detection.

In evaluating the relation between oral contraceptive use and HPV infection, it may be appropriate to include, where possible, studies using different methods of HPV detection as these may reflect different aspects of HPV infection. Serological studies are thought to measure cumulative lifetime exposure to HPV (although it is not clear to what extent persistence of infection is reflected in antibody response), whereas measures of cervical HPV DNA are likely to reflect current and persistent past infection (Shin *et al*, 2003). Non-PCR methods of detecting HPV DNA may preferentially detect infections of high viral load and these may be of particular clinical significance (Ylitalo *et al*, 2000; Lorincz *et al*, 2002).

The relation between oral contraceptive use and the use of barrier methods of contraception is a potential source of confounding, and it has not been possible to address this here. In some populations, nonusers of oral contraceptives are likely to use barrier methods, and as many as a third of young oral contraceptive users in some recent studies report also using condoms (Peipert *et al*, 1997; Svare *et al*, 1997). Since barrier contraceptives may reduce the risk of HPV transmission (Manhart and Koutsky, 2002), such associations may be important.

Most eligible studies included some women with cytological abnormalities; while the number of women with severe grades of cytological abnormality (cervical intraepithelial neoplasia (CIN) 3, high-grade squamous cell intraepithelial lesions (HSIL) or above) was very small (typically about 1%), the strong association between cervical cancer and HPV means that they accounted for up to 10% of the HPV-positive women in these studies. This seems unlikely to prove a significant source of bias in the measured relation between oral contraceptive use and HPV. Low-grade cytological abnormalities (CIN1/2, LSIL), which may be present in up to 10% of women in these unselected populations, and which may account for a further 10% of HPV positives in these populations, may simply indicate HPV infection and should probably not be considered a possible source of bias.

There are a number of hypotheses but little direct evidence about the ways in which oral contraceptives might influence cervical HPV infection. The use of oral contraceptives is associated with an increased incidence of cervical ectropion, which means that the site where HPV infection preferentially induces neoplastic lesions, the squamo-columnar junction, is more exposed to potential carcinogens. Oestrogen and progestogens may also affect cervical cells directly, increasing cell proliferation and stimulating transcription of HPVs (de Villiers, 2003). Oral contraceptives might thus affect not only viral infection and malignant transformation of cervical cells but also the sensitivity or specificity of tests for detecting HPV infection.

In conclusion, this review provides preliminary evidence suggesting that there is no strong positive or negative relation between oral contraceptive use and prevalent infection of the cervix with HPV. Given that HPV infection appears to be a necessary cause of most cases of cervical cancer, this finding, if true, is of considerable importance as it suggests that HPV status is not likely to confound examination of the relation between long duration use of oral contraceptives and the risk of cervical cancer. However, there are serious problems in interpreting these results and further evidence is needed to address the methodological and other issues raised.
